# Characteristics of malaria vector populations and transmission before a randomised controlled trial assessing the efficacy of next-generation insecticide-treated nets in Côte d’Ivoire

**DOI:** 10.1186/s13071-025-06921-w

**Published:** 2025-07-10

**Authors:** Ludovic Phamien Ahoua Alou, Alphonsine Amanan Koffi, Edouard Dangbenon, Soromane Camara, Joseph Biggs, Marius Gonse Zoh, Serge Brice Assi, Colette Sih, Benoit Talbot, Manisha Ann Kulkarni, Louisa Alexandra Messenger, Natacha Protopopoff, Jackie Cook, Raphael N’Guessan

**Affiliations:** 1 Institut Pierre Richet (IPR)/Institut National de Santé Publique (INSP), Bouaké, Côte d’Ivoire; 2 Vector Control Product Evaluation Centre (VCPEC-IPR/INSP), Bouaké, Côte d’Ivoire; 3 Centre d’Entomologie Médicale et Vétérinaire, Université Alassane Ouattara (CEMV-UAO), Bouaké, Côte d’Ivoire; 4 Faculty of Epidemiology and Population Health, Department of Infectious Disease Epidemiology, London School of Hygiene and Tropical Medicine, London, WC1E 7HT UK; 5 School of Epidemiology & Public Health, Faculty of Medicine, University of Ottawa, Ottawa, ON Canada; 6 Faculty of Infectious and Tropical Diseases, Disease Control Department, London School of Hygiene and Tropical Medicine, London, WC1E 7HT UK; 7 Department of Environmental and Occupational Health, School of Public Health, University of Nevada, Las Vegas, NV 89154 USA; 8 Health Interventions Unit, Department of Epidemiology and Public Health, Swiss Tropical & Public Health Institute, Kreuzstrasse 2, 4123 Allschwill, Switzerland; 9 University of Basel, Basel, Switzerland; 10 Medical Research Council (MRC) International Statistics and Epidemiology Group, Department of Infectious Disease Epidemiology and International Health, London School of Hygiene and Tropical Medicine, London, WC1E 7HT UK

**Keywords:** *Anopheles gambiae* s.l., *Anopheles funestus*, Malaria transmission, Côte d’Ivoire

## Abstract

**Background:**

The World Health Organization (WHO) recommends mass distribution of insecticide-treated nets (ITNs) to prevent malaria transmission. Unfortunately, resistance to pyrethroids affects the efficacy of standard ITNs. To overcome this resistance and continue to protect the population, the WHO has recommended new types of ITNs that combine a pyrethroid insecticide with either a synergist (PBO) or a second insecticide, such as chlorfenapyr. This study examines the baseline characteristics of malaria vectors prior to the distribution of three types of insecticide-treated nets as part of a three-arm randomised controlled trial: Interceptor G2 (pyrethroid–chlorfenapyr), VEERALIN (pyrethroid–PBO), and MAGNet (pyrethroid only).

**Methods:**

The study was carried out in 40 villages (grouped into 33 clusters) of Tiébissou district in central Côte d’Ivoire. To assess biting rate and biting behaviour, human landing catches were conducted hourly indoors and outdoors in six randomly selected houses in each cluster, starting at 18:00 and continuing until 08:00 the next morning. Adult mosquitoes collected were morphologically identified, and a subset of *Anopheles gambiae* sensu lato (s.l.) and *An. funestus* s.l. were speciated by quantitative PCR (qPCR). *Plasmodium* sporozoite infections were detected by qPCR to estimate infection rates. The entomological inoculation rate was calculated as the product of the mosquito biting rate and the sporozoite infection rate.

**Results:**

Among the 10,698 mosquitoes collected, *An. gambiae* s.l. was the predominant species, accounting for 62.5% (*n* = 6683) of the catch, followed by *An. funestus* s.s., which accounted for 19.8% (*n* = 2120). Of the sub-sample of *An. gambiae* s.l. processed by PCR, 79.0% (*n* = 1291/1635) were *An. coluzzii* and the remaining were *Anopheles gambiae* s.s. Malaria vectors were highly aggressive, with an average of 14.8 bites/person/night for *An. coluzzii*, 2.0 b/p/n for *An. gambiae* s.s. and 5.4 b/p/n for *An. funestus*, representing an overall average of 22.2 b/p/n (95% CI 17.2–27.2 b/p/n). No significant difference was found in biting activity between indoor and outdoor environments (*Z* = −0.25, *P* = 0.803). *Plasmodium* sporozoite infection rate was 2.4% (95% CI 1.3–3.6%) for *An. coluzzii*, 1.5% (95% CI 0.3–2.6%) for *An. gambiae* s.s. and 2.7% (95% CI 1.2–4.3%) for *An. funestus*. The estimated overall entomological inoculation rate was 0.4 infected b/p/n (95% CI 0.3–0.6) and varied between 0.0 and 0.2 infective bites/person/night according to species. There was no difference observed in entomological infection rate (EIR) between capture locations (indoors versus outdoors; *Z* = 1.521, *P* = 0.128).

**Conclusions:**

This study shows that *An. coluzzii* and *An. funestus* were the main malaria vectors and showed similar biting patterns both indoors and outdoors. *Anopheles funestus* was found in high density in a limited number of villages. Malaria transmission was high despite universal distribution of pyrethroid-ITN in the district.

**Graphical Abstract:**

**Figure:**
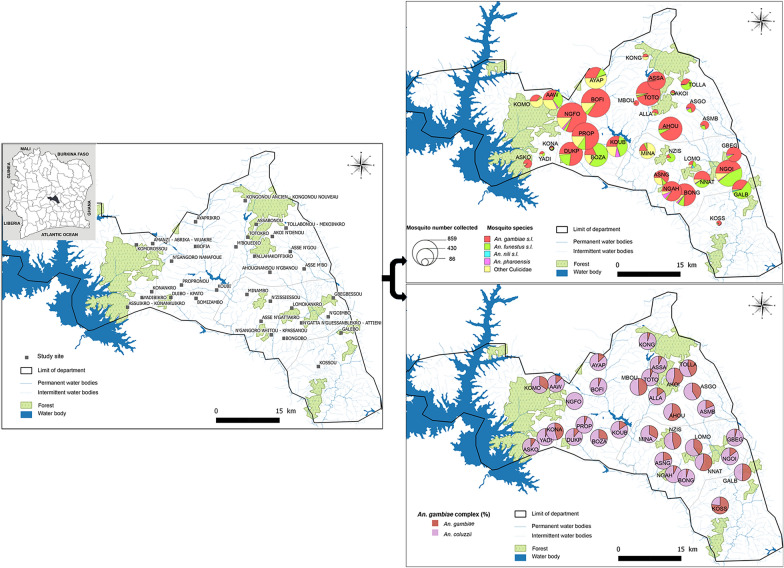

**Supplementary Information:**

The online version contains supplementary material available at 10.1186/s13071-025-06921-w.

## Background

Malaria is a vector-borne disease caused by the *Plasmodium* parasite, transmitted to humans through the bite of infected female *Anopheles* mosquitoes. Malaria burden in Côte d’Ivoire is a significant public health concern and remains a major cause of morbidity and mortality, particularly among children under 5 years old and pregnant women [1]. *Plasmodium falciparum* is responsible for most malaria cases in Côte d’Ivoire. The primary vectors in Côte d’Ivoire are *Anopheles gambiae* sensu stricto (s.s.), *An. coluzzii* and *An. funestus* sensu lato (s.l.) [2–7]. Another vector, *An. nili* s.l. has also been found to play a key role in local transmission [5, 8].

Even though malaria occurs throughout Côte d’Ivoire, malaria transmission dynamics are heterogeneous between regions depending on environmental factors such as ecological settings, climatic factors, agricultural practices and human behaviour [9–12]. In addition to these factors, vector biology and behaviour, such as host feeding activity, biting cycles and host preferences, also influence malaria transmission. For example, changes in mosquito biting behaviour have been observed following the deployment of vector control tools inside houses [13]. Monitoring such changes is important for understanding the parameters affecting malaria epidemiology and vector dynamics and for applying the most effective vector control strategies.

To prevent malaria transmission and reduce morbidity and mortality, the Côte d’Ivoire National Malaria Control Programme (NMCP) has implemented various strategies, including mass distribution of pyrethroid insecticide-treated nets (ITNs), chemoprevention, diagnostic testing, and treatment. Between 2000 and 2015, these strategies reduced malaria cases and deaths by 33% and 68% in Côte d’Ivoire, respectively. However, since 2015, there has been no further reduction in the mortality rate but rather an increase in the number of cases in 2023. The last mass distribution campaign of ITNs took place in 2021, during which nearly 19 million ITNs were distributed. However, malaria incidence remained high in 2023, with more than 7.8 million cases, with almost 1452 deaths reported, for an expected 9900 deaths [1]. ITNs are central in the prevention and control of malaria, but to be successful, they need to be effective against local malaria vectors. Unfortunately, resistance to pyrethroids, the most common chemical used in long-lasting insecticidal nets (LLINs), is widespread in malaria vectors [14–16], and impacts the efficacy of standard pyrethroid ITNs [17, 18]. To overcome this resistance and continue to protect people, the WHO has recommended new types ITNs that combine a pyrethroid insecticide with either a synergist (PBO) or a second insecticide, such as chlorfenapyr [1]. These recommendations are based on a limited number of trials conducted in Tanzania [19, 20], Uganda [21] and Benin [22]. The need for convincing data on the effectiveness of new tools in areas of high resistance to insecticides remains necessary to demonstrate their added benefit in reducing the incidence and prevalence of malaria. For example, there is no evidence on the epidemiological efficacy of the combination of PBO and pyrethroid nets against malaria from West Africa. Information on the impact of these nets on malaria transmission in these West African settings will also generate important evidence for their efficacy.

This study examines the baseline characteristics of malaria vectors in Tiebissou, Côte d’Ivoire, prior to the distribution of three types of insecticide-treated nets: Interceptor^®^ G2 (pyrethroid–chlorfenapyr), VEERALIN^®^ (pyrethroid–PBO), and MAGNet^®^ (pyrethroid only). This research is part of a randomised controlled trial (RCT) evaluating the efficacy of these nets for reducing malaria transmission and incidence.

## Methods

### Study site and design

**Fig. 1:**
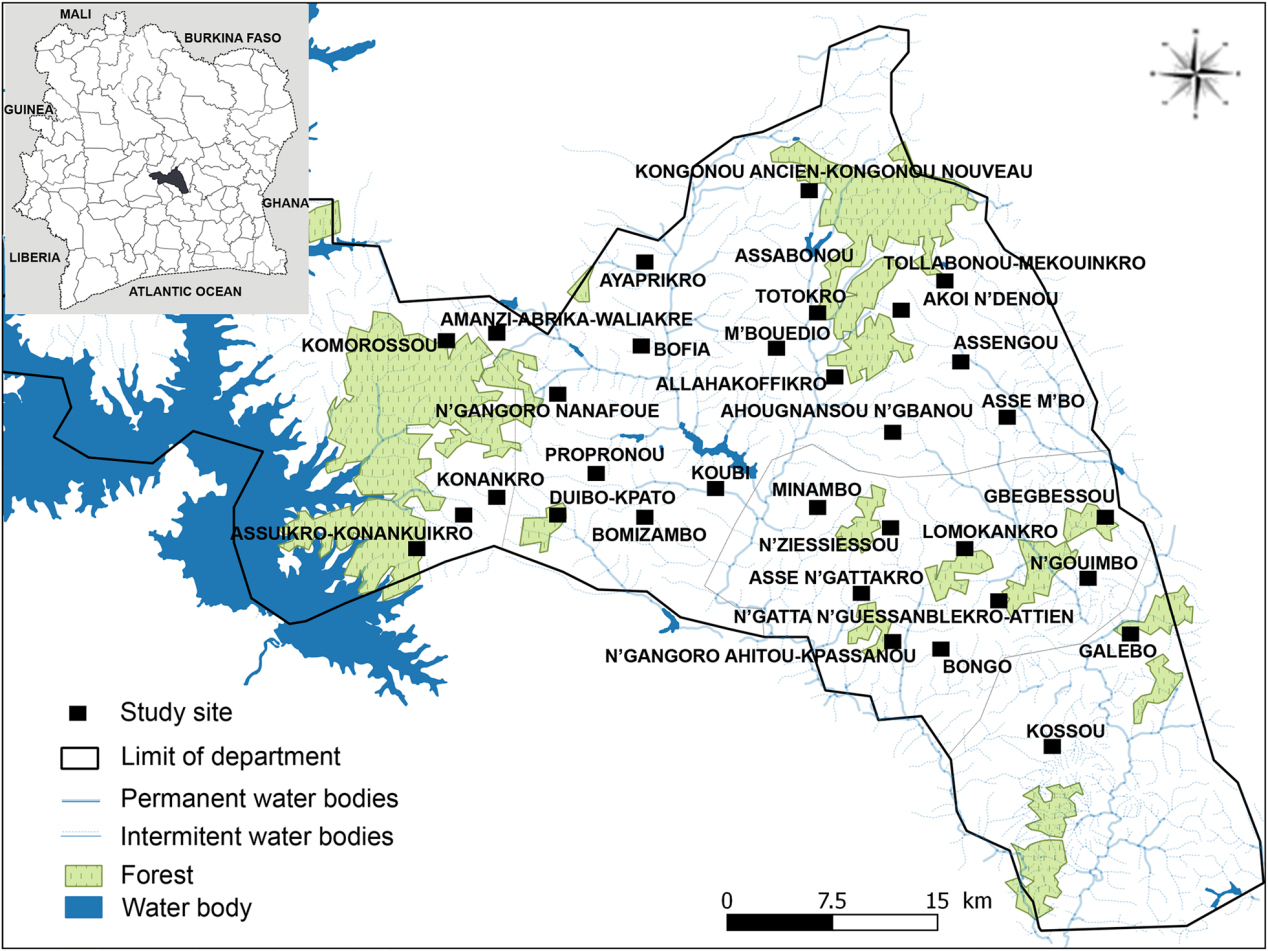
Map of Tiébissou district showing the location of villages used for entomological collection. Insert: map of Côte d’Ivoire showing the location of Tiébissou district

The study was carried out in the district of Tiébissou (7°09’ N, 5°14’ W; Fig. 1) in central Côte d’Ivoire (Belier region), about 40 km north of Yamoussoukro (political capital city of Côte d’Ivoire) and within 60 km of Bouake (second largest town in Côte d’Ivoire). The area is characterised by intense malaria transmission with over half of children under 5 infected in 2021 (51.3%) [23]. Intense pyrethroid resistance associated with the almost fixed (≥ 80%) 995F *kdr* mutation, and overexpression of cytochrome P450 genes have been found in *An. gambiae* s.l. [16, 24, 25]. The natural vegetation is mainly wooded savannah and pre-forest savannah. There are typically two rainy periods (March–July and September–October), with an average annual rainfall of approximately 800 mm and an average temperature of 26.6 °C. Agriculture is the main economic activity, dominated by cash crops (cocoa and cashew) and food crops such as cassava, yam and rice.

The study took place in 40 villages (grouped into 33 clusters; population approximately 7476) which were selected on the basis of their accessibility during the rainy season, a size of 200–500 inhabitants per cluster and a distance between each cluster of at least 2 km (Fig. 1). According to a census carried out by our team in June 2023, i.e. 2 years after a government-led mass distribution campaign, the proportion of households with at least one ITN was 66.2%, and with at least one ITN for every two inhabitants was 16.5%.

### Mosquito collection and determination

Between July and August 2023, one round of mosquito collections using human landing catches (HLC) was undertaken in six randomly selected households in all clusters. HLCs involved a trained volunteer, sitting on a stool with their legs exposed. Collectors wore shorts or rolled-up trousers and clothing that covered their bodies and upper limbs to protect them from unnecessary mosquito bites. Mosquitoes landing on the legs were collected hourly using small glass tubes plugged with cotton [26] and stored in labelled collection bags. Four collectors working in pairs were required per house. Collections were done within randomly selected inhabited houses. Outdoor collection was carried out within a radius of 5–10 m around the selected house and the distance between selected houses was at least 200 m apart. Two collectors (one indoor and another outdoor) collected mosquitoes between 18:00 to 01:00 and the second group from 01:00 to 08:00 The two collectors of the same group swapped from indoors to outdoors every 2 h to account for any possible differences in individual attractiveness to mosquitoes. Supervisors visited each house every night to ensure quality of the work and rotation of the collectors.

### Morphological identification

Female *Anopheles* mosquitoes collected were morphologically identified to species level using a key for Afrotropical *Anopheles* mosquitoes [27]. Due to the large numbers of *Anopheles* spp. captured, a subsample of 30% from indoor and outdoor collections and across all collection hours was randomly selected from each cluster and stored in individual tubes with silica gel and preserved for further analysis. From this subsample, a subset of at least 50 females (when more than 50 were collected) with 1–4 individuals of *An. gambiae* s.l. or *An. funestus* s.l. per hour per collection site (indoors, outdoors) were randomly selected for molecular analysis.

### Molecular analysis

The head–thorax of morphologically identified *An. gambiae* s.l. and *An. funestus* s.l. was used for genomic DNA extraction using the cetyl trimethyl ammonium bromide (CTAB) 2% method described in Yahouedo et al. [28]. Genomic DNA extracted from individual *Anopheles* spp. was used to detect *Plasmodium* infection using a quantitative polymerase chain reaction (qPCR) assay [29], slightly modified. A total reaction volume of 10 µl contained 2 µl of HOT FIREPol EvaGreen qPCR Mix Plus (no ROX) 5× (Solis Biodyne, Tartu, Estonia), 0.3 µl of each of PL1473F18 (5′-TAA CgA ACg AgA TCT TAA-3′) and PL1679R18 (5′-gTT CCT CTA AgA AgC TTT-3′) primers (diluted 1/10), 6.4 µl of nuclease-free PCR-grade water and 1 µl of mosquito genomic DNA template. Amplification started with an initial denaturation step at 95 °C for 12 min, followed by 50 cycles of denaturation at 95 °C for 10 s, annealing at 50 °C for 5 s and elongation at 72 °C for 20 s, with release of fluorescence at the end of each cycle. A final amplification step is carried out at 95 °C for 30 s and at 65 °C for 30 s, followed by a temperature increase from 0.2 °C/s to 95 °C with fluorescence acquisition at each temperature. Melting curves generated at different temperatures allowed identification of each *Plasmodium* species: *P. malariae*: 74.3–75.4 °C, *P. falciparum*: 75.5–77.2 °C and *P. ovale*: 77.5–79.5 °C.

The sibling species of the *An. gambiae* complex, namely *An. coluzzii*, *An. gambiae* s.s. and *An. arabiensis*, were distinguished using a qPCR method [30] with a universal forward primer (SINE200Fa 5′-ATTGCTACCACCAAAATACATGAAA-3′), a reverse primer matching both *An. coluzzii* and *An. gambiae* with G-8 extension (SINE200Rd5′-GGGGGGGGGAATAATAAGGAACTGCATTTAAT-3′), and an *An. arabiensis* specific reverse primer (SINE200Re5′- GGATGTCTAATAGTCTCAATAGATG -3′). Melting curves generated at different temperatures allowed us to identify each *An. gambiae* sibling species: *An. coluzzii*: > 85 °C, *An. gambiae*: 74–85 °C and *An. arabiensis*: < 74 °C. Members of the *An. funestus* group were distinguished by a published qPCR (TaqMan) method to identify *An. funestus* s.s., *An. leesoni*, *An. parensis*, *An. rivulorum* and *An. vaneedeni* [31]. Melting curves generated at different temperatures allowed us to identify each *An. funestus* sibling species.

### Household data collection

A questionnaire on household characteristics and ITN ownership and use was administered in the households where HLCs were conducted using Open Data Kit (ODK). Information recorded included the number of inhabitants in the surveyed households, the number of people sleeping indoors and outdoors, the type of nets presents in the house, the number of people sleeping under nets, other malaria prevention measures used by household members, and the GPS coordinates of the households.

### Parameters measured

Entomological indicators of malaria transmission measured both indoors and outdoors were the following:

- 1. Human biting rate (HBR), which indicated the number of bites per person per night (b/p/n), was calculated by dividing the number of mosquitoes collected by the number of collectors per night during the sampling period:
  $$HBR \, = \, Total \, mosquitoes \, collected/number \, of \, collectors \, by \, night$$.
- 2. Sporozoite infection rate (SIR) was estimated as the proportion of anopheline mosquitoes positive for *Plasmodium* sporozoites over the total number of mosquitoes tested: (number of anopheline mosquitoes positive for *Plasmodium* sporozoites in the head–thorax/total number of anopheline mosquitoes tested) × 100.
- 3. Entomological inoculation rate (EIR), which indicated the number of infected bites per human per night (ib/h/n) for each *Anopheles* species and for overall *Anopheles* spp., was determined as follows: *EIR* = *(HBR × SIR)/100.*

### Data analysis

Household characteristics were collected using the Open Data Kit (ODK) during the night of capture. Information on mosquitoes (morphological identification of species and sex) were recorded on paper before being entered twice with CsPro.7.2, and then cleaned and analysed in Stata 15.0 (Stata Corp., College Station, TX). Descriptive analysis of household and species characteristics was performed. Analysis was done at the cluster level and by capture location (indoor and outdoor). Entomological indicators (HBR, SIR and EIR) were presented by species (*An. gambiae* s.l. and *An. funestus* s.l.) and both combined. Differences in HBR, SIR and EIR indoors versus outdoors, and between malaria species, were analysed by Wilcoxon test for paired samples and Mann–Whitney test for independent samples (after assessment of non-normal distribution and homogeneity of data by the Shapiro–Wilk test and the Levene test, respectively). These tests were performed with a *P*-value < 0.05 to determine the statistical significance of the differences observed.

## Results

### Household and individual characteristics of the study population

A total of 198 households with 1796 inhabitants were visited for HLC, with an average household size of between 9 and 10 persons (Table 1). Within these households, 77.1% (95% CI 75.1–79.0%) of the people slept indoors and the majority of houses were made of cement (80.1%; 95% CI 73.9–85.2%). More than half of the households (52.0%; 95% CI 45.0–59.0%) reported owning at least one net, and the proportion of people that reported sleeping under nets the previous night was 26.3% (95% CI 24.3–28.4%).

**Table 1:** Household and individual characteristics of the study population

Indicators	Study area (*N* or mean and 95% CI)
Total number of clusters	33
Households	
Total number of households	10,630
Total number of visited households	196
Total number of people in households	1796
Median number of people per visited household (IQR)	8.0 (5–11)
Type of housing	
Cement wall	80.1%
Mud wall	10.2%
Cement + mud wall	9.7%
ITNs	
Total number of ITNs within the visited households	372
Proportion of visited household with at least one ITN (95% CI)	52.0% (45.0–59.0)
Median number of ITNs per household (IQR)	3 (2–5)
Proportion of people sleeping under ITN the previous night in the visited household	*N* = 472 (26.8%; 95% CI 24.7–28.9%)

*N* number of, *95% CI* 95% confidence interval, *ITN* insecticide treated net

### Mosquito species composition and densities

**Fig. 2:**
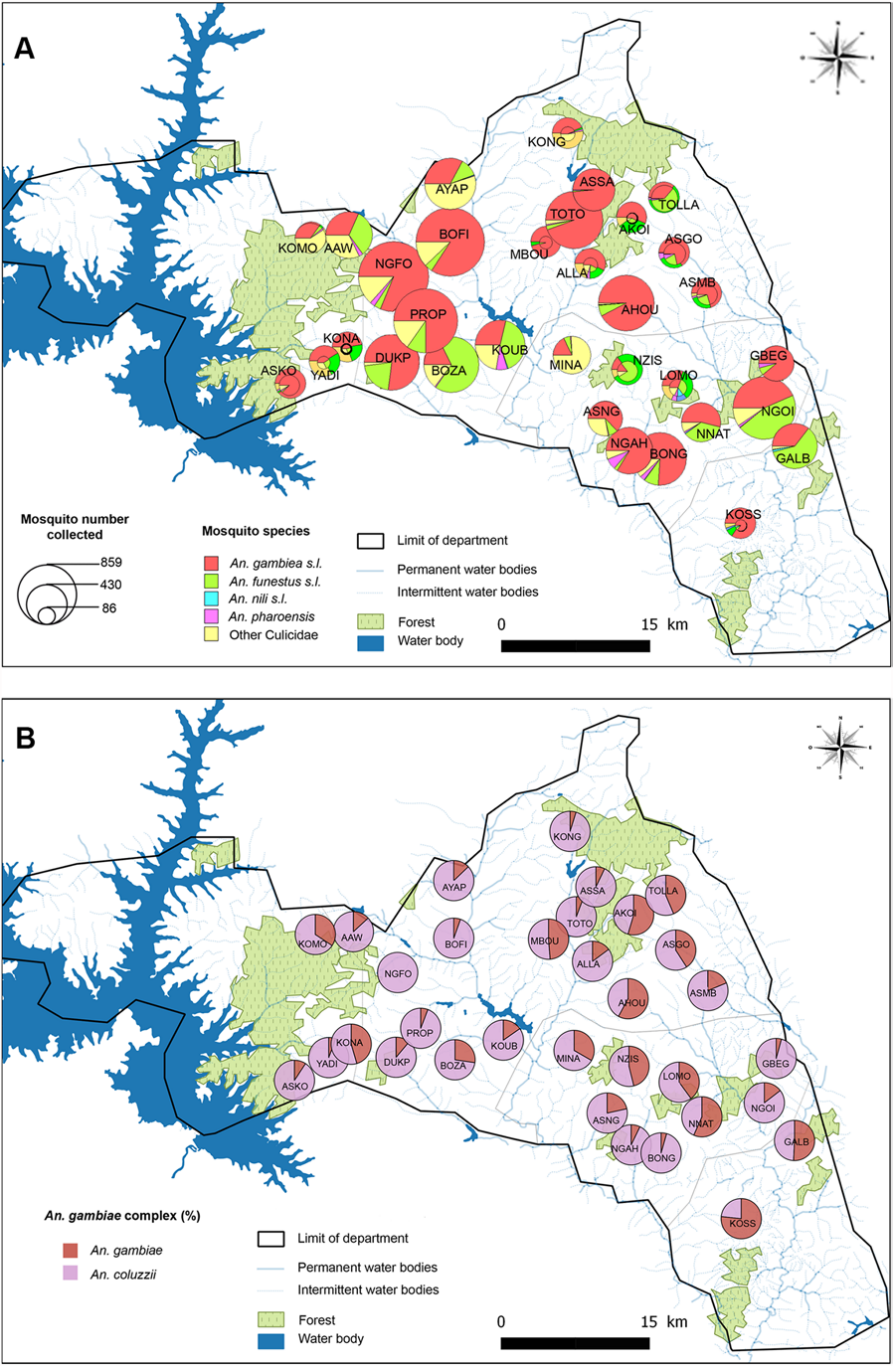
Map of mosquito densities and composition in the 33 clusters of the study area. **A** Overall mosquito density. **B** *Anopheles gambiae* s.l. sibling species distribution. *AAW* Amanzi-Abrika-Wuakre, *AHOU* Ahougnansou N’Ganou, *AKOI* Akoi N’Denou, *ALLA* Allahakoffikro, *ASGO* Asse N’Gou, *ASKO* Assuikro-Konankuikro, *ASMB* Asse M’Bo, *ASNG* Asse N’Gattakro, *ASSA* Assabonou, *AYAP* Ayaprikro, *BOFI* Bofia, *BONG* Bongobo, *BOZA* Bomizambo, *DUKP* Duibo-Kpato, *GALB* Galebo, *GBEG* Gbegbessou, *KOMO* Komorossou, *KONA* Konankro, *KONG* Kongonou Ancien-Kongonou Nouveau, *KOSS* Kossou, *KOUB* Koubi, *LOMO* Lomokankro, *MBOU* M’Bouedio, *MINA* Minambo, *NGAH* N’Gangoro Ahitou-Kpassanou, *NGFO* N’Gangoro Nanafoue, *NGOI* N’Goimbo, *NNAT* N’Gatta N’Guessanblekro-Attienkoffikro, *NZIS* N’Zissiessou, *PROP* Proponou, *TOLLA* Tollabonou-Mekoinkro, *TOTO* Totokro, *YADI* Yadibikro, *An. Anopheles*

A total of 396 person-nights of collection were conducted. A total of 10,698 mosquitoes belonging to four genera were collected: *Anopheles* spp. (*n* = 9031, 84.4%), *Mansonia* spp. (*n* = 1071, 10.0%), *Culex* spp. (*n* = 551, 5.2%) and *Aedes* spp. (*n* = 45, 0.4%) (Table 2). Among the 9031 *Anopheles* mosquitoes morphologically identified, 62.5% (*n* = 6683) were members of the *An. gambiae* complex, 19.8% (*n* = 2120) belonged to the *An. funestus* group, and 0.4% (*n* = 41) were members of *An. nili* complex. *Anopheles gambiae* s.l. was, by far, the most abundant mosquito collected in the study area (Fig. 2A). The density of *An. nili* s.l. was too low (*n* = 40, 0.3%) for a clear pattern to be observed, and for this reason, it has not been considered in the rest of the analyses.

**Table 2:** Composition and abundance of mosquitoes collected by human-landing catch (HLC) in Tiébissou District

Genera	Species	All		Location			
				Indoors		Outdoors	
		*N*	%^a^	*N*	%^b^	*N*	%^b^
*Anopheles*	*An. gambiae* s.l.	6683	62.5	3146	47.1	3537	52.9
	*An. funestus* s.l.	2120	19.8	1134	53.5	986	46.5
	*An. nili* s.l.	41	0.4	11	26.8	30	73.2
	*An. pharoensis*	187	1.8	57	30.5	130	69.5
*Culex*	*Cx. quinquefasciatus*	265	2.5	103	38.9	162	61.1
	*Cx. nebulosus*	140	1.3	62	44.3	78	55.7
	*Cx. annulioris*	122	1.1	65	53.3	57	46.7
	*Cx. tigripes*	9	0.1	3	33.3	6	66.7
	*Cx. poicilipes*	15	0.1	1	6.7	14	93.3
*Aedes*	*Ae. aegypti*	44	0.4	12	27.3	32	72.7
	*Ae. africanus*	1	0.0	0	0.0	1	100
*Mansonia*	*Ma. africana*	965	9.0	285	29.5	680	70.5
	*Ma. uniformis*	106	1.0	33	31.1	73	68.9

^a^Number of mosquitoes collected expressed in percentage of the total number of mosquitoes collected (%) in the study area

^b^Number of mosquitoes collected expressed in percentage of the total number of mosquitoes collected (%) indoor and outdoor

Among the 1635 Anopheles individuals morphologically identified as members of the *An. gambiae* complex, 79.0% (*n* = 1291) were *An. coluzzii* and the remaining were *An. gambiae* s.s. (21.0%; *n* = 344). The relative abundance and species composition of the *An. gambiae* s.l. population varied from one cluster to another (Fig. 2A and B). All *An. funestus* s.l. morphologically identified and successfully analysed by PCR (*n* = 1443) were all *An. funestus* s.s. (henceforth simply referred to as *An. funestus*).

### Biting patterns of malaria vectors

**Fig. 3:**
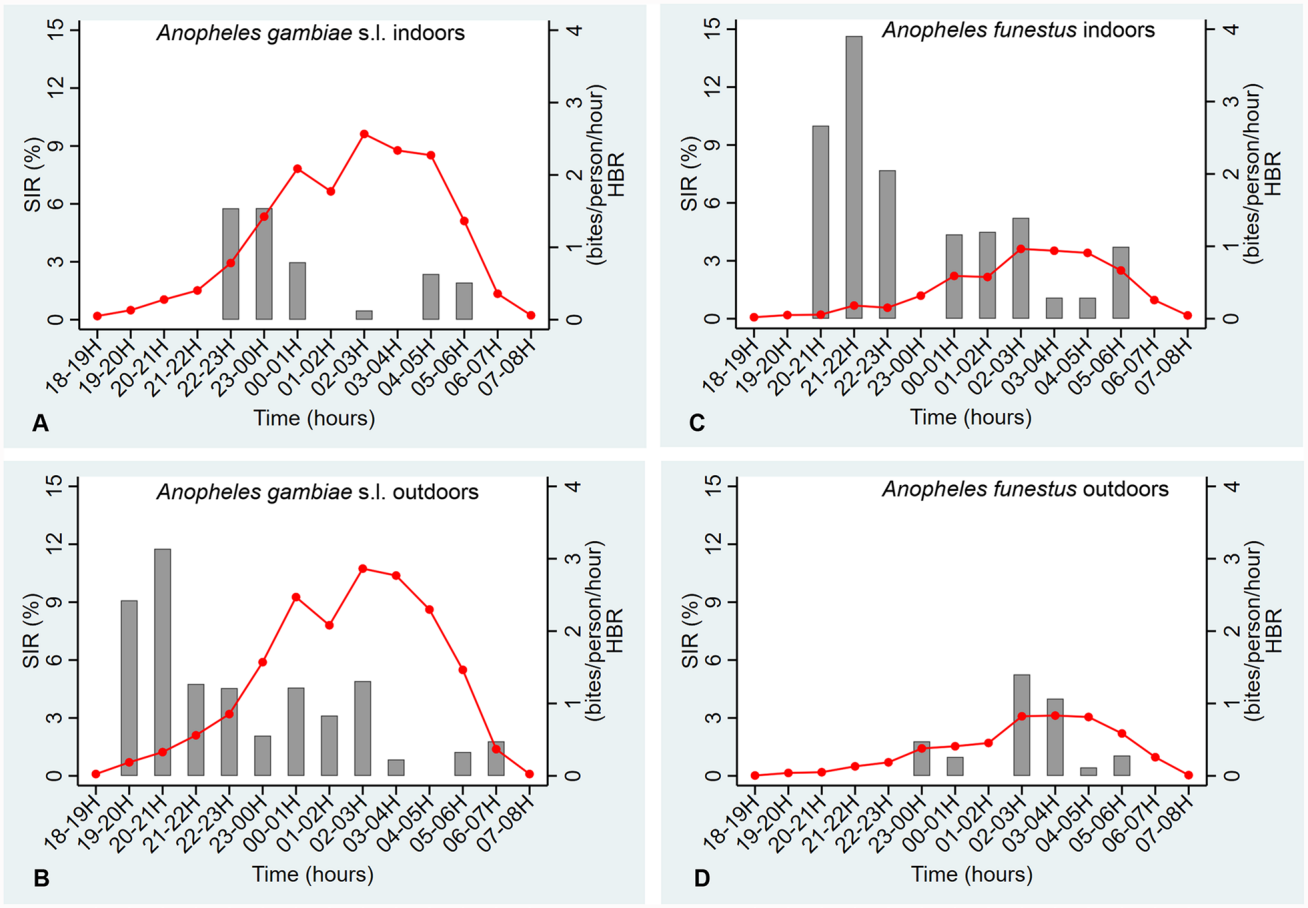
Indoor (**A**, **C**) and outdoor (**B**, **D**) mean hourly human biting patterns of *Anopheles gambiae* s.l. (**A**, **B**) and *An. funestus* (**C**, **D**) in relation to average hourly sporozoite infection rate (SIR) in the Tiébissou area. Human biting rate (HBR) is expressed as the number of bites received per person and per hour. Sporozoite infection rate (SIR) is expressed as a percentage of infected mosquitoes per hour. The grey bar represents the SIR per hour

The pattern of biting activities of *An. gambiae* s.l. and *An. funestus* by hour are shown in Fig. 3, with biting occurring during all hours of collection. The biting activity was observed to increase gradually from dusk, then peaking between 00:00 and 05:00 before decreasing towards dawn. Indoor (Fig. 3A) and outdoor (Fig. 3B) biting activities of *An. gambiae* s.l. were relatively similar, with greater activity occurring in the second half of the night with peaks between 02:00 and 03:00. The night biting activity profiles of *An. funestus* (Fig. 3C and D) were similar to that of *An. gambiae* s.l. With *An. funestus*, increased biting activity also occurred in the second half of the night with a peak seen from 02:00 to 03:00 indoors, and from 02:00 to 05:00 outdoors.

Figure 3 shows the biting time of infected mosquitoes. The indoor biting activity of infected *An. gambiae* s.l. was bimodal, the early and higher peak, with 70% of infected bites, happening between 22:00 and 01:00 with the smaller peak of infected bites occurring between 04:00 and 06:00 (25%). The outdoor biting activity of infected *An. gambiae* s.l. was observed from dusk to dawn compared with indoor, with three peaks: 19:00–21:00 (40%), 02:00–03:00 (12%) and the last in early morning, 06:00–07:00 (12%). Infected *An. funestus* were found to bite earlier in the evening, with 59% of infected bites occurring between 20:00 and 23:00 indoors, whilst outdoors, infected *An. funestus* bites were more evenly distributed throughout the night.

### Human biting rates (HBRs)

**Fig. 4:**
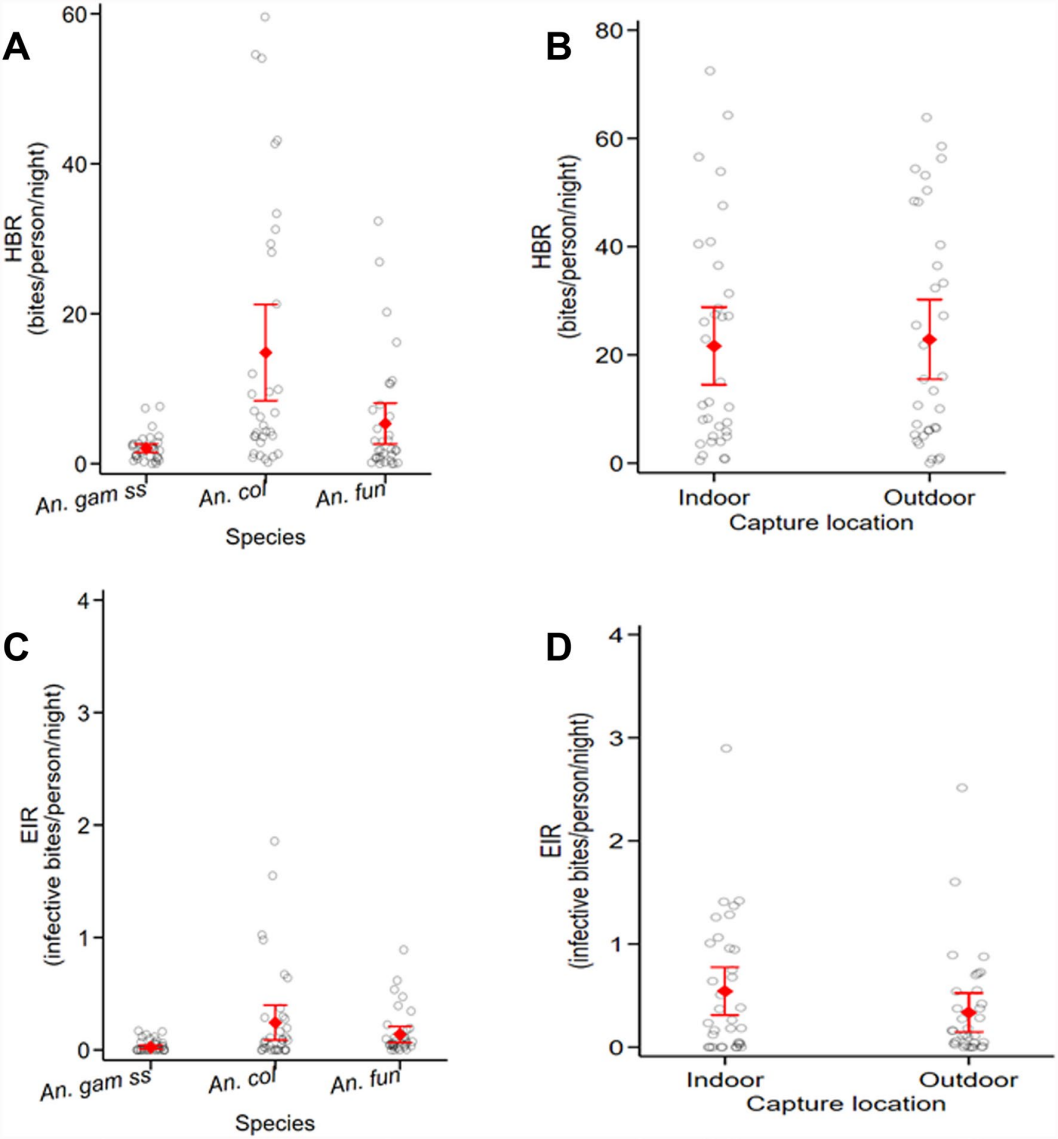
Human biting rate (HBR; **A**, **B**) and entomological inoculation rate (EIR; **C**, **D**) of malaria vector species in the Tiébissou area. Results are presented as means and 95% CIs. Small circles indicate HBR or EIR recorded at each cluster and red dots show the mean HBR or EIR, the red bars indicate the 95% confidence intervals. Human biting rate (HBR) is expressed as the number of bites received per person and per night. Entomological inoculation rate (EIR) is expressed as a number of infective bites received per person and per night. *An. gam ss*, *Anopheles gambiae* s.s.; *An. col*, *An. coluzzii*; *An. fun*, *An. funestus*

The HBR of malaria vectors varied according to species and capture location (Fig. 4A; Table 3). For *An. coluzzii* the HBR indoors was 14.1 bites/person/night (b/p/n) and 15.5 b/p/n outdoors. No difference was found between capture locations (*Z* = −0.199, *P* = 0.842), and the trend was similar for *An. gambiae* s.s. (*Z* = −1.715, *P* = 0.087) and *An. funestus* (*Z* = −0.559, *P* = 0.581). The overall average biting rate of *An. funestus* (5.4 b/p/n, 95% CI 3.4–7.3 b/p/n) was significantly lower than *An. gambiae* s.l. (*An. coluzzii* + *An. gambiae s.s.*) (16.9 b/p/n, 95% CI 12.3–21.5 b/p/n) (*Z* = 4.593, *P* < 0.001).

**Table 3:** Summary of the entomological parameters of malaria transmission in the study area of Tiébissou

Index	Capture location		*An. gambiae* s.s.	*An. coluzzii*	*An. gambiae* s.l.*	*An. funestus* s.s.	All vectors
HBR (b/p/n)	Indoors	Mean [95% CI] median (Q25–Q75)	1.8 [1.1–2.5] 1.1 (0.4–2.3)	14.1 [7.5–20.7] 5.0 (1.3–21.8)	15.9 [9.2–22.5] 7.7 (2.8–23.3)	5.7 [2.8–8.7] 2.5 (0.9–6.7)	21.6 [14.4–28.8] 12.0 (4.6–33.6)
	Outdoors	Mean [95% CI] median (Q25–Q75)	2.3 [1.6–3.0] 2.0 (0.9–3.1)	15.5 [9.1–22.0] 4.9 (2.6–31.5)	17.9 [11.2–24.5] 7.3 (3.4–32.8)	5.0 [2.4–7.6] 1.3 (0.7–6.6)	22.8 [15.5–30.2] 13.5 (4.7–44.0)
	Total	Mean [95% CI] median (Q25–Q75)	2.0 [1.6–2.5] 1.6 (0.5–2.7)	14.8 [10.3–19.3] 4.9 (1.8–24.1)	16.9 [12.3–21.5] 7.5 (2.8–28.0)	5.4 [3.4–7.3] 2.0 (0.8–6.5)	22.2 [17.2–27.2] 13.4 (4.8–36.4)
SIR (%)	Indoors	Mean [95% CI] median (Q25–Q75)	1.4 [0.0–3.1] 0.0 (0.0–0.0)	1.4 [0.3–2.5] 0.0 (0.0–1.4)	1.6 [0.6–2.6] 0.0 (0.0–2.9)	3.3 [1.4–5.3] 0.0 (0.0–4.9)	2.5 [1.6–3.4] 2.0 (0.0–4.1)
	Outdoors	Mean [95% CI] median (Q25–Q75)	1.5 [0.0–3.2] 0.0 (0.0–0.0)	3.5 [1.4–5.6] 0.0 (0.0–6.2)	2.9 [1.3–4.6] 0.0 (0.0–5.5)	2.1 [0.0–4.7] 0 (0.0–0.0)	2.9 [1.4–4.5] 1.0 (0.0–4.6)
	Total	Mean [95% CI] median (Q25–Q75)	1.5 [0.3–2.6] 0.0 (0.0–0.0)	2.4 [1.2–3.6] 0.0 (0.0–3.4)	2.3 [1.3–3.2] 0.0 (0.0–3.5)	2.7 [1.2–4.3] 0.0 (0.0–2.8)	2.7 [1.9–3.6] 1.9 (0.0–4.1)
EIR (ib/p/n)	Indoors	Mean [95% CI] median (Q25–Q75)	0.0 [0.0–0.0] 0.0 (0.0–0.0)	0.2 [0.0–0.5] 0.0 (0.0–0.1)	0.3 [0.0–0.5] 0.0 (0.0–0.2)	0.22 [0.09–0.34] 0.00 (0.00–0.26)	0.6 [0.3–0.8] 0.3 (0.0–1.0)
	Outdoors	Mean [95% CI] median (Q25–Q75)	0.0 [0.0–0.0] 0.0 (0.0–0.0)	0.2 [0.0–0.4] 0.0 (0.0–0.3)	0.3 [0.1–0.5] 0.0 (0.0–0.3)	0.06 [0.01–0.11] 0.00 (0.00–0.00)	0.4 [0.2–0.5] 0.2 (0.0–0.5)
	Total	Mean [95% CI] median (Q25–Q75)	0.0 [0.0–0.0] 0.0 (0.0–0.0)	0.2 [0.1–0.4] 0.0 (0.0–0.2)	0.3 [0.1–0.4] 0.0 (0.0–0.3)	0.13 [0.07–0.21] 0.00 (0.00–0.20)	0.5 [0.3–0.6] 0.2 (0.0–0.7)

^*^An. gambiae s.l. corresponding to *An. gambiae* s.s. + *An. coluzzii*

Human biting rate (HBR) expressed as a number of bites of malaria vectors received per person and per night; mean and corresponding 95% confidence interval (95% CI), and median and corresponding interquartile range (IQR) 25th and 75th percentiles. Sporozoite infection rate (SIR) expressed as a number of mosquitoes infected with *Plasmodium* sp. sporozoites per 100 mosquitoes; mean and corresponding 95% confidence interval (95% CI), and median and corresponding interquartile range (IQR) 25th and 75th percentiles. Entomological inoculation rate (EIR) for *Plasmodium* sp. expressed as a number of infected bites received per person and per night; mean and corresponding 95% confidence interval (95% CI), and median and corresponding interquartile range (IQR) 25th and 75th percentiles

The overall (indoor and outdoor combined) average human biting rate (HBR) of *Anopheles* malaria vectors (*An. gambiae* s.l. and *An. funestus*) was 22.2 bites/person/night (b/p/n) (95% CI 17.2–27.2 b/p/n) in the study area (Fig. 4B; Table 3). No difference was found between the mean HBR indoors (21.6 b/p/n; 95% CI 14.4–28.8 b/p/n) and outdoors (22.8 b/p/n; 95% CI 15.5–30.2 b/p/n) (*Z* = −0.25, *P* = 0.803). Similar trends were observed with both species. The detailed HBR per study cluster are shown in Additional file 1: Table 1 and Additional file 2: Table 2.

### Sporozoite infection rates (SIRs)

In total, 3071 head–thoraxes of *Anopheles* mosquitoes, comprising 1285 *An. coluzzii*, 342 *An. gambiae* s.s. and 1444 *An. funestus*, were tested for the presence of *Plasmodium* spp. in salivary glands. The majority of the parasites detected were *P. falciparum* (90.1%, *n* = 64), followed by *P. malariae* (7.0%, *n* = 5) and *P. ovale* (2.8%, *n* = 1). The sporozoite infection rates (SIR) for the different *Anopheles* malaria vectors are shown in Table 3. 

No significant differences in SIR were detected between *An. coluzzii* (2.4%, 95% CI 1.2–3.6%) and *An. gambiae* s.s. (1.5%, 95% CI 0.3–2.6%) (*Z* = −1.536, *P* = 0.125), nor between mosquitoes collected indoors and outdoors (indoors: 1.4% *An. gambiae* s.s., 1.4% *An. coluzzii*, *Z* = −0.752, *P* = 0.452; outdoors: 1.5% *An. gambiae* s.s., 3.5% *An. coluzzii*, *Z* = −1.420, *P* = 0.156) (Table 3). Thus, there was no variation in average SIR between indoors and outdoors for *An. gambiae* s.l. (*Z* = −0.988, *P* = 0.323). Out of 1627 *An. gambiae* s.l. tested, an overall 1.6% (95% CI 0.6–2.6%) SIR was estimated. The overall SIR for *An. funestus* was 2.7% (95% CI 1.2–4.3%), with a significant difference detected between indoor (3.3%, 95% CI 1.4–5.3%) and outdoor (2.1%, 95% CI 0.0–4.7%) SIRs (*Z* = 2.157, *P* = 0.031). There was no significant difference in the overall SIRs for *An. gambiae* s.l. and *An. funestus* (*Z* = 0.045, *P* = 0.964) (Table 3). Of these, 70 specimens were infected, representing an overall SIR of 2.7% (95% CI 1.9–3.6%) for all clusters. Combined data from all malaria vectors (*An. coluzzii*, *An. gambiae* s.s. and *An. funestus*) showed a similar proportion of infections indoors (2.5%, 95% CI 1.6–3.4%) and outdoors (2.9%, 95% CI 1.4–4.5%) (*Z* = 0.658, *P* = 0.511). The detailed SIRs per study cluster are shown in Additional file 1: Table 1 and Additional file 2: Table 2.

### Entomological inoculation rates (EIRs)

The entomological inoculation rates (EIRs) are shown in Fig. 4C and D; Table 3; Additional file 1: Table 1 and Additional file 2: Table 2. No differences in EIRs were detected between indoors and outdoors (0.02 infective bite per person per night [ib/p/n]) for *An. gambiae* ss. The same trend was observed for *An. coluzzii* (indoors and outdoors EIR was 0.2 ib/p/n). Overall, the estimated EIR of *An. gambiae* s.l. was the same indoors and outdoors (0.3 ib/p/n). For *An. funestus*, the overall average EIR was 0.1 (95% CI 0.1–0.2) ib/p/n and was higher indoors than outdoors (*Z* = 2.207, *P* = 0.027). No significant difference in EIR was detected between *An. gambiae* s.l. and *An. funestus* (*Z* = 0.639, *P* = 0.523).

The overall average EIR for all vector species combined was 0.5 (95% CI 0.3–0.6) infective bites per person per night (ib/p/n) in the study area. No difference was observed between capture locations (indoors: 0.6 (95% CI 0.3–0.8) ib/p/n, outdoors: 0.4 (95% CI 0.2–0.5) ib/p/n; *Z* = 1.541, *P* = 0.123).

## Discussion

The main objective of this study was to characterise the malaria vector species composition, abundance, behaviour, and infectivity, as well as malaria transmission, prior to the implementation of an RCT assessing the efficacy of next-generation ITNs on malaria. The vectors identified in this study were mostly from the *An. gambiae* complex (*An. coluzzii* and *An. gambiae* s.s.) and *An. funestus*, with *An. coluzzii* being the predominant species. Peak biting activity occurred between 01:00 and 03:00. In this study, *An. coluzzii*, *An. gambiae* s.s. and *An. funestus* had similar *Plasmodium* infection rates, ranging from 1.4% to 3.5%, depending on location (outdoor/indoor) and species. The high vector density and EIR confirm the significant risk of malaria exposure for populations living in the Tiébissou district.

The presence of *An. gambiae* s.l. and *An. funestus* in the Tiébissou district with varying abundance and proportion from one village (cluster) to another was consistent with past studies in Côte d’Ivoire [2–7]. In the present study, a survey of larval habitats was not carried out to assess the presence of potential breeding sites in and around the villages; however, the contrast in diversity and abundance of vectors collected could give some indications on the diversity and abundance of larval habitats within the Tiébissou area. Local ecological contrasts (geographical and hydrographic) and disparity in the distribution of breeding sites from cluster to cluster could explain, at least partially, these differences in abundance. The two sibling species of *An. gambiae* s.l. found were *An. coluzzii* and *An. gambiae* s.s., with *An. coluzzii* being the dominant species. These two sibling species show close association with breeding habitats close to human dwellings [32]. The wide distribution and predominance of *An. coluzzii* suggests a more favourable environment for its proliferation and greater adaptability to multiple settings. In West Africa, a correlation between *An. coluzzii* and *An. gambiae* s.s. and ecological factors has been shown, indicating that *An. coluzzii* prefers to breed in permanent and semi-permanent man-made aquatic habitats, while temporary breeding aquatic habitats created by rain are favourable for *An. gambiae* s.s. [33, 34]. *Anopheles funestus* was found in all the villages in the Tiébissou area. The Tiébissou area (tropical climate), mostly characterised by wooded and pre-forest savannahs with greater exposure to sunlight, and the presence of numerous aquatic habitats with vegetation are known to be favourable to *An. funestus* breeding. In various studies, *An. funestus* was found to breed in permanent and semi-permanent aquatic habitats with vegetation and stagnant or slow-moving waters, such as river streams and swamps [35–37]. The heterogeneity of abundance of *An. funestus* in the area was clear, with high density found in 4 out of 33 clusters, with proportions ranging from 51–78.6% of the collected mosquitoes. These clusters were within the immediate vicinity of large water bodies with upright vegetation.

Within this study, no difference in biting rate was observed between indoor and outdoor collections with *An. gambiae* s.l. or *An. funestus*. Endophagy is usually the expected dominant behaviour in *An. gambiae* s.l. and *An. funestus* mosquito populations, but these results suggest no preference for endophagic or exophagic behaviour, in line with previous reports from Côte d’Ivoire [38, 39]. Other studies have also shown that *An. gambiae* s.l. and *An. funestus* were either endophagic or exophagic [2, 40]. The endophagic or exophagic status would suggest a plasticity or opportunistic biting behaviour linked to host availability. In the case of HLC, for example, it cannot be ruled out that modifying human behaviour by placing a host indoors or outdoors for the duration of the capture session may induce bias that could also modify mosquito host seeking behaviour. As expected, sporozoite-positive *An. gambiae* s.l. and *An. funestus* were detected both indoors and outdoors. However, surprisingly, there seemed to be more infected mosquitoes captured between 20:00 and 22:00 both indoors for *An. funestus*, and outdoors for *An. gambiae* s.l. at the time of the night where vector densities were low, and people not yet sleeping under ITNs. This trend of early biting of infected females indoors and outdoors is relevant and may suggest a change in behaviour of infected females to avoid ITNs. Previous studies have indicated that infection can modify Anopheline feeding behaviour, such as increasing attractiveness to human odours [41] and enhanced probing time when feeding. Changes in the host-seeking behaviour of malaria vectors *An. gambiae* s.l. and *An. funestus* have also been found in other settings after scaling up universal coverage of ITNs [13, 42, 43]. These changes show the ability of these vectors (especially infected females) to adapt their host-seeking behaviour to the timing of human activities and sleeping behaviour, and ultimately to circumvent vector control interventions that limit human–vector contact. However, one should be prudent with the interpretation of the early infected bites, as a limited number of Anopheline mosquitoes were collected during the early hours of the study, which could be a major limitation. Further studies with larger mosquito sample sizes are needed to confirm these findings.

Aggregated data in the study area shows a high overall infection rate in *An. gambiae* s.l. and *An. funestus*, and no significant differences between capture locations. These observed results are consistent with previous studies in nearby areas in Côte d’Ivoire [38, 39]. Of concern is the fraction of infected *An. gambiae* s.l. and *An. funestus* observed biting early outdoors and indoors when people are not under ITNs, as it would contribute to maintaining malaria transmission despite the presence of ITNs within the home. This malaria transmission occurring outside common vector activity times and places, and maintained despite coverage of a population with ITNs, is referred to as residual malaria transmission [44]. Outdoor and early evening or morning malaria transmission is regarded as the main cause of residual malaria transmission, representing a key challenge across all malaria-endemic countries that prompts the need to implement integrated vector management control strategies. Continuing to use conventional ITNs and indoor residual spraying (IRS), which only target endophagous and endophilic vectors, will have limited impact on outdoor biting mosquitoes.

## Conclusions

The entomological indicators in the Tiébissou setting have shown that malaria transmission was high, with mainly *P. falciparum* transmitted by *An. gambiae* s.s., *An. coluzzii* and *An. funestus*. While *An. coluzzii* was the predominant vector, the presence of a high proportion of infected *An. funestus* was indicative of them being an efficient vector despite low densities. A majority of the infectious bites by both *An. gambiae* s.l. and *An. funestus* occurred in early evening, indoors and outdoors, when people are unprotected by ITNs. This suggests that additional vector control measures are necessary to complement the deployment of ITNs.

## Supplementary Information

Additional file 1. Table S1. Entomological indicators of malaria transmission (human biting, sporozoite infection and entomological inoculation rates) for *An. gambiae *s.l. per study cluster

Additional file 2. Table S2. Entomological indicators of malaria transmission (human biting, sporozoite infection and entomological inoculation rates) for *An. funestus* per study cluster

## Data Availability

Data supporting the conclusions of this article are included within this article and its additional files. For further information, the dataset used and analysed are available on reasonable request from the VCPEC-IPR in Côte d’Ivoire.

## Abbreviations

CI: Confidence interval
EIR: Entomological infection rate
HLC: Human landing catch
HBR: Human biting rate
ITN: Insecticide treated net
IRS: Indoor residual spraying
LLIN: Long-lasting insecticidal net
b/p/n: Bites per person per night
ib/p/n: Infective bites per person per night
OR: Odds ratio
PCR: Polymerase chain reaction
SIR: Sporozoite infection rate
