# Supplementary material for: Characteristics of malaria vector populations and transmission before a randomised controlled trial assessing the efficacy of next-generation insecticide-treated nets in Côte d’Ivoire
Source: Parasit Vectors. 2025 Jul 10;18:277. doi: 10.1186/s13071-025-06921-w (PMC12247193; doi:10.1186/s13071-025-06921-w)
Supplement: Supplementary file 1 — Additional file 1. Table S1. Entomological indicators of malaria transmission (human biting, sporozoite infection and entomological inoculation rates) for An. gambiae s.l. per study cluster [file 13071_2025_6921_MOESM1_ESM.docx]

**Additional file 1: Table 1.** Entomological indicator of malaria transmission (human biting, sporozoite infection and entomological inoculation rates) per study cluster for *An. gambiae* s.l.

| Cluster | Capture location | | | | | | | | | | Overall | | | | |
| --- | --- | --- | --- | --- | --- | --- | --- | --- | --- | --- | --- | --- | --- | --- | --- |
|  | Indoor | | | | | Outdoor | | | | |  |  |  |  |  |
|  | N | Mean HBR  (95% CI) | Inf./Tested | SIR | EIR | N | Mean HBR (95% CI) | Inf./Tested | SIR | EIR | N | Mean HBR  (95% CI) | Inf./Tested | SIR | EIR |
| TOTO | 246 | 41.0 (36.2-45.8) | 3/35 | 8.6 | 3.51 | 316 | 52.7 (42.1-63.2) | 0/35 | 0.0 | 0.00 | 562 | 46.8 (40.5-53.2) | 3/70 | 4.3 | 1.76 |
| MBOU | 13 | 2.2 (-0.4-4.7) | 0/12 | 0.0 | 0.00 | 29 | 4.8 (1.3-8.4) | 0/27 | 0.0 | 0.00 | 42 | 3.5 (0.9-6.1) | 0/39 | 0.0 | 0.00 |
| ASSA | 163 | 27.2 (20.2-34.2) | 0/35 | 0.0 | 0.00 | 192 | 32.0 (25.0-39.0) | 0/36 | 0.0 | 0.00 | 355 | 29.6 (23.7-35.5) | 0/71 | 0.0 | 0.00 |
| AKOI | 9 | 1.5 (0.0-3.0) | 0/5 | 0.0 | 0.00 | 9 | 1.5 (0.5-2.5) | 0/6 | 0.0 | 0.00 | 18 | 1.5 (0.4-2.6) | 0/11 | 0.0 | 0.00 |
| ASGO | 46 | 7.7 (1.4-13.9) | 0/22 | 0.0 | 0.00 | 33 | 5.5 (1.9-9.1) | 2/22 | 9.1 | 0.50 | 79 | 6.6 (3.2-10.0) | 2/44 | 4.5 | 0.25 |
| TOLLA | 22 | 3.7 (2.6-4.8) | 0/15 | 0.0 | 0.00 | 35 | 5.8 (2.2-9.4) | 2/24 | 8.3 | 0.49 | 57 | 4.8 (2.6-6.9) | 2/39 | 5.1 | 0.24 |
| ASMB | 29 | 4.8 (1.9-7.8) | 1/21 | 4.8 | 0.23 | 42 | 7.0 (4.4-9.6) | 3/27 | 11.1 | 0.78 | 71 | 5.9 (3.3-8.5) | 4/48 | 8.3 | 0.50 |
| GBEG | 329 | 54.8 (47.3-62.4) | 0/36 | 0.0 | 0.00 | 357 | 59.5 (52.2-66.8) | 0/35 | 0.0 | 0.00 | 686 | 57.2 (52.4-61.9) | 0/71 | 0.0 | 0.00 |
| NGOI | 118 | 19.7 (11.5-27.8) | 0/24 | 0.0 | 0.00 | 182 | 30.3 (26.5-34.2) | 0/31 | 0.0 | 0.00 | 300 | 25.0 (19.9-30.1) | 0/55 | 0.0 | 0.00 |
| NNAT | 81 | 13.5 (9.4-17.6) | 0/27 | 0.0 | 0.00 | 87 | 14.5 (8.2-20.8) | 0/26 | 0.0 | 0.00 | 168 | 14.0 (9.2-18.8) | 0/53 | 0.0 | 0.00 |
| ALLA | 16 | 2.7 (1.1-4.3) | 0/15 | 0.0 | 0.00 | 17 | 2.8 (0.9-4.7) | 0/12 | 0.0 | 0.00 | 33 | 2.8 (1.3-4.2) | 0/27 | 0.0 | 0.00 |
| AHOU | 18 | 3.0 (-0.3-6.3) | 0/19 | 0.0 | 0.00 | 42 | 7.0 (5.0-9.0) | 1/38 | 2.6 | 0.18 | 60 | 5.0 (3.2-6.8) | 1/57 | 1.8 | 0.09 |
| NZIS | 10 | 1.7 (0.8-2.6) | 0/10 | 0.0 | 0.00 | 4 | 0.7 (0.1-1.3) | 0/3 | 0.0 | 0.00 | 14 | 1.2 (0.5-1.9) | 0/13 | 0.0 | 0.00 |
| GALB | 65 | 10.8 (5.4-16.3) | 0/32 | 0.0 | 0.00 | 74 | 12.3 (5.8-18.9) | 1/33 | 3.0 | 0.37 | 139 | 11.6 (5.9-17.2) | 1/65 | 1.5 | 0.19 |
| ASNG | 80 | 13.3 (6.8-19.9) | 1/37 | 2.7 | 0.36 | 75 | 12.5 (4.5-20.5) | 3/42 | 7.1 | 0.89 | 155 | 12.9 (6.1-19.8) | 4/79 | 5.1 | 0.63 |
| BONG | 125 | 20.8 (15.5-26.2) | 1/38 | 2.6 | 0.55 | 265 | 44.2 (33.5-54.8) | 1/30 | 3.3 | 1.47 | 390 | 32.5 (25.2-39.8) | 2/68 | 2.9 | 1.01 |
| KONG | 8 | 1.3 (0.0-2.6) | 0/8 | 0.0 | 0.00 | 6 | 1.0 (0.3-1.7) | 1/6 | 16.7 | 0.17 | 14 | 1.2 (0.7-1.7) | 1/14 | 7.1 | 0.08 |
| AYAP | 34 | 5.7 (0.6-10.7) | 0/17 | 0.0 | 0.00 | 125 | 20.8 (11.9-29.7) | 0/22 | 0.0 | 0.00 | 159 | 13.3 (7.0-19.5) | 0/39 | 0.0 | 0.00 |
| BOFI | 360 | 60.0 (39.2-80.8) | 1/32 | 3.1 | 1.88 | 330 | 55.0 (23.6-86.4) | 0/42 | 0.0 | 0.00 | 690 | 57.5 (43.3-71.7) | 1/74 | 1.4 | 0.94 |
| KOMO | 34 | 5.7 (0.3-11.1) | 1/16 | 6.3 | 0.35 | 33 | 5.5 (1.6-9.4) | 0/19 | 0.0 | 0.00 | 67 | 5.6 (2.1-9.0) | 1/35 | 2.9 | 0.18 |
| BOZA | 42 | 7.0 (4.1-9.9) | 0/27 | 0.0 | 0.00 | 57 | 9.5 (6.8-12.2) | 0/28 | 0.0 | 0.00 | 99 | 8.3 (6.2-10.3) | 0/55 | 0.0 | 0.00 |
| KONA | 2 | 0.3 (0.0-0.7) | 0/2 | 0.0 | 0.00 | 9 | 1.5 (-0.2-3.2) | 0/8 | 0.0 | 0.00 | 11 | 0.9 (-0.1-2.0) | 0/10 | 0.0 | 0.00 |
| DUKP | 185 | 30.8 (15.4-46.2) | 1/26 | 3.8 | 1.19 | 255 | 42.5 (26.5-58.5) | 0/28 | 0.0 | 0.00 | 440 | 36.7 (23.2-50.2) | 1/54 | 1.9 | 0.59 |
| PROP | 283 | 47.2 (27.5-66.9) | 0/25 | 0.0 | 0.00 | 264 | 44.0 (14.6-73.4) | 0/27 | 0.0 | 0.00 | 547 | 45.6 (27.5-63.7) | 0/52 | 0.0 | 0.00 |
| NGFO | 414 | 69.0 (34.2-103.8) | 0/36 | 0.0 | 0.00 | 283 | 47.2 (34.0-60.3) | 2/33 | 6.1 | 2.86 | 697 | 58.1 (34.8-81.3) | 2/69 | 2.9 | 1.43 |
| ASKO | 63 | 10.5 (4.8-16.2) | 0/33 | 0.0 | 0.00 | 24 | 4.0 (1.5-6.5) | 0/21 | 0.0 | 0.00 | 87 | 7.3 (3.5-11.0) | 0/54 | 0.0 | 0.00 |
| YADI | 7 | 1.2 (0.3-2.0) | 0/7 | 0.0 | 0.00 | 11 | 1.8 (0.9-2.8) | 1/11 | 9.1 | 0.17 | 18 | 1.5 (0.8-2.2) | 1/18 | 5.6 | 0.08 |
| LOMO | 10 | 1.7 (1.1-2.3) | 1/10 | 10.0 | 0.17 | 15 | 2.2 (1.2-3.1) | 2/15 | 13.3 | 0.29 | 25 | 1.9 (1.4-2.5) | 3/25 | 12.0 | 0.23 |
| AAW | 87 | 14.5 (10.6-18.4) | 0/37 | 0.0 | 0.00 | 44 | 7.3 (5.5-9.1) | 0/24 | 0.0 | 0.00 | 131 | 10.9 (8.5-13.4) | 0/61 | 0.0 | 0.00 |
| KOUB | 55 | 9.2 (6.4-11.9) | 2/40 | 5.0 | 0.46 | 78 | 13.0 (8.7-17.3) | 1/45 | 2.2 | 0.29 | 133 | 11.1 (8.5-13.7) | 3/85 | 3.5 | 0.37 |
| MINA | 21 | 3.5 (0.8-6.2) | 0/10 | 0.0 | 0.00 | 30 | 5.0 (2.9-7.1) | 1/20 | 5.0 | 0.25 | 51 | 4.3 (2.3-6.2) | 1/30 | 3.3 | 0.13 |
| NGAH | 154 | 25.7 (16.0-35.3) | 0/58 | 0.0 | 0.00 | 201 | 33.5 (23.4-43.6) | 0/54 | 0.0 | 0.00 | 355 | 29.6 (22.5-36.6) | 0/112 | 0.0 | 0.00 |
| KOSS | 17 | 2.8 (0.1-5.5) | 1/16 | 6.3 | 0.18 | 15 | 2.5 (1.2-3.8) | 0/14 | 0.0 | 0.00 | 32 | 2.7 (0.9-4.5) | 1/30 | 3.3 | 0.09 |

HBR: human biting rate expressed as number of b/p/n; SIR: sporozoite infection rate expressed in percent; EIR: entomological rate expressed as number of infected bites/p/n; CI: confidence interval; N: number of mosquitoes collected; Inf./Tested: number of mosquitoes found infected with *Plasmodium* spp. over the total number of mosquitoes tested

Cluster: AAW, Amanzi-Abrika-Wuakre; AHOU, Ahougnansou N’Ganou; AKOI, Akoi N’Denou; ALLA, Allahakoffikro; ASGO, Asse N’Gou; ASKO, Assuikro-Konankuikro; ASMB, Asse M’Bo; ASNG, Asse N’Gattakro; ASSA, Assabonou; AYAP, Ayaprikro; BOFI, Bofia; BONG, Bongobo; BOZA, Bomizambo; DUKP, Duibo-Kpato; GALB, Galebo; GBEG, Gbegbessou; KOMO, Komorossou; KONA, Konankro; KONG, Kongonou Ancien-Kongonou Nouveau; KOSS, Kossou; KOUB, Koubi; LOMO, Lomokankro; MBOU, M’Bouedio; MINA, Minambo; NGAH, N’Gangoro Ahitou-Kpassanou; NGFO, N’Gangoro Nanafoue; NGOI, N’Goimbo; NNAT, N’Gatta N’Guessanblekro-Attienkoffikro; NZIS, N’Zissiessou; PROP, Proponou; TOLLA, Tollabonou-Mekoinkro; TOTO, Totokro; YADI, Yadibikro
