# Supplementary material for: Characteristics of malaria vector populations and transmission before a randomised controlled trial assessing the efficacy of next-generation insecticide-treated nets in Côte d’Ivoire
Source: Parasit Vectors. 2025 Jul 10;18:277. doi: 10.1186/s13071-025-06921-w (PMC12247193; doi:10.1186/s13071-025-06921-w)
Supplement: Supplementary file 2 — Additional file 2. Table S2. Entomological indicators of malaria transmission (human biting, sporozoite infection and entomological inoculation rates) for An. funestus per study cluster [file 13071_2025_6921_MOESM2_ESM.docx]

**Additional file 2: Table 2.** Entomological indicators of malaria transmission (human biting, sporozoite infection and entomological inoculation rates) per study cluster for *An. funestus*

| Cluster | Capture location | | | | | | | | | | | Overall | | | | |  |
| --- | --- | --- | --- | --- | --- | --- | --- | --- | --- | --- | --- | --- | --- | --- | --- | --- | --- |
|  | Indoor | | | | | Outdoor | | | | | |  |  |  |  |  |  |
|  | N | Mean HBR  (95% CI) | Inf./Tested | SIR | EIR | | N | Mean HBR  (95% CI) | Inf./Tested | SIR | EIR | N | Mean HBR  (95% CI) | Inf./Tested | SIR | EIR | |
| TOTO | 9 | 1.5 (0.2-2.8) | 0/8 | 0.0 | 0.00 | | 5 | 0.8 (0.1-1.6) | 0/5 | 0.0 | 0.00 | 14 | 1.2 (0.4-1.9) | 0/13 | 0.0 | 0.00 | |
| MBOU | 1 | 0.2 (-0.1-0.5) | 0/1 | 0.0 | 0.00 | | 1 | 0.2 (-0.1-0.5) | 0/1 | 0.0 | 0.00 | 2 | 0.2 (0.0-0.4) | 0/2 | 0.0 | 0.00 | |
| ASSA | 1 | 0.2 (-0.1-0.5) | 0/1 | 0.0 | 0.00 | | 2 | 0.3 (0.0-0.7) | 0/2 | 0.0 | 0.00 | 3 | 0.3 (-0.1-0.6) | 0/3 | 0.0 | 0.00 | |
| AKOI | 5 | 0.8 (0.1-1.6) | 0/4 | 0.0 | 0.00 | | 5 | 0.8 (-0.3-2.0) | 0/5 | 0.0 | 0.00 | 10 | 0.8 (-0.1-0.7) | 0/9 | 0.0 | 0.00 | |
| ASGO | 16 | 2.7 (-0.1-5.4) | 0/15 | 0.0 | 0.00 | | 7 | 1.2 (0.3-2.0) | 0/7 | 0.0 | 0.00 | 23 | 1.9 (0.5-2.3) | 0/22 | 0.0 | 0.00 | |
| TOLLA | 50 | 8.3 (3.9-12.7) | 1/45 | 2.2 | 0.19 | | 45 | 7.5 (3.0-12.0) | 0/35 | 0.0 | 0.00 | 95 | 7.9 (3.8-8.1) | 1/80 | 1.3 | 0.09 | |
| ASMB | 9 | 1.5 (0.0-3.0) | 0/6 | 0.0 | 0.00 | | 15 | 2.5 (0.8-4.2) | 0/9 | 0.0 | 0.00 | 24 | 2.0 (0.7-3.3) | 0/15 | 0.0 | 0.00 | |
| GBEG | 12 | 2.0 (0.5-3.5) | 1/7 | 14.3 | 0.29 | | 12 | 2.0 (0.3-3.7) | 0/10 | 0.0 | 0.00 | 24 | 2.0 (0.5-3.5) | 1/17 | 5.9 | 0.14 | |
| NGOI | 166 | 27.7 (17.1-38.3) | 2/74 | 2.7 | 0.75 | | 147 | 24.5 (19.7-29.3) | 1/70 | 1.4 | 0.35 | 313 | 26.1 (19.8-32.4) | 3/144 | 2.1 | 0.55 | |
| NNAT | 71 | 11.8 (5.9-17.7) | 2/45 | 4.4 | 0.53 | | 40 | 6.7 (4.2-9.2) | 2/39 | 5.1 | 0.34 | 111 | 9.3 (6.3-12.2) | 4/84 | 4.8 | 0.43 | |
| ALLA | 8 | 1.3 (0.2-2.4) | 0/6 | 0.0 | 0.00 | | 2 | 0.3 (0.0-0.7) | 0/2 | 0.0 | 0.00 | 10 | 0.8 (0.1-1.6) | 0/8 | 0.0 | 0.00 | |
| AHOU | 15 | 2.5 (0.1-4.9) | 0/11 | 0.0 | 0.00 | | 13 | 2.2 (-0.7-5.0) | 0/13 | 0.0 | 0.00 | 28 | 2.3 (0.8-3.9) | 0/24 | 0.0 | 0.00 | |
| NZIS | 45 | 7.5 (6.0-9.0) | 2/37 | 5.4 | 0.41 | | 29 | 4.8 (1.4-8.3) | 0/25 | 0.0 | 0.00 | 74 | 6.2 (4.3-8.0) | 2/62 | 3.2 | 0.20 | |
| GALB | 126 | 21.0 (6.2-35.8) | 4/67 | 6.0 | 1.25 | | 107 | 17.8 (10.7-25.0) | 1/54 | 1.9 | 0.33 | 233 | 19.4 (9.0-29.9) | 5/121 | 4.1 | 0.79 | |
| ASNG | 15 | 2.5 (-0.6-5.6) | 0/12 | 0.0 | 0.00 | | 6 | 1.0 (-0.1-2.1) | 0/4 | 0.0 | 0.00 | 21 | 1.8 (-0.4-3.9) | 0/16 | 0.0 | 0.00 | |
| BONG | 20 | 3.3 (0.6-6.0) | 0/12 | 0.0 | 0.00 | | 26 | 4.3 (1.8-6.9) | 0/25 | 0.0 | 0.00 | 46 | 3.8 (1.3-6.4) | 0/37 | 0.0 | 0.00 | |
| KONG | 1 | 0.2 (-0.1-0.5) | 0/1 | 0.0 | 0.00 | | 0 | - | - | - | - | 1 | 0.1 (-0.1-0.2) | 0/1 | 0.0 | 0.00 | |
| AYAP | 15 | 2.5 (1.2-3.8) | 0/14 | 0.0 | 0.00 | | 39 | 6.5 (1.8-11.2) | 0/35 | 0.0 | 0.00 | 54 | 4.5 (1.9-7.1) | 0/49 | 0.0 | 0.00 | |
| BOFI | 22 | 3.7 (0.8-6.5) | 0/20 | 0.0 | 0.00 | | 8 | 1.3 (-0.3-2.9) | 0/8 | 0.0 | 0.00 | 30 | 2.5 (0.7-4.3) | 0/28 | 0.0 | 0.00 | |
| KOMO | 6 | 1.0 (0.5-1.5) | 1/5 | 20.0 | 0.20 | | 2 | 0.3 (-0.3-0.9) | 0/2 | 0.0 | 0.00 | 8 | 0.7 (0.5-0.9) | 1/7 | 14.3 | 0.10 | |
| BOZA | 201 | 33.5 (18.6-48.4) | 3/103 | 2.9 | 0.98 | | 181 | 30.2 (22.2-38.1) | 0/97 | 0.0 | 0.00 | 382 | 31.8 (22.3-41.4) | 3/200 | 1.5 | 0.49 | |
| KONA | 1 | 0.2 (-0.1-0.5) | 0/1 | 0.0 | 0.00 | | 4 | 0.7 (-0.1-1.4) | 1/4 | 25.0 | 0.17 | 5 | 0.4 (0.1-0.8) | 1/5 | 20.0 | 0.08 | |
| DUKP | 27 | 4.5 (0.9-8.1) | 0/27 | 0.0 | 0.00 | | 93 | 15.5 (9.3-21.7) | 3/85 | 3.5 | 0.55 | 120 | 10.0 (7.2-12.8) | 3/112 | 2.7 | 0.27 | |
| PROP | 35 | 5.8 (-0.9-12.5) | 1/25 | 4.0 | 0.23 | | 36 | 6.0 (1.0-11.0) | 0/32 | 0.0 | 0.00 | 71 | 5.9 (2.2-9.7) | 1/57 | 1.8 | 0.12 | |
| NGFO | 18 | 3.0 (0.6-5.4) | 1/17 | 5.9 | 0.18 | | 7 | 1.2 (0.2-2.1) | 0/6 | 0.0 | 0.00 | 25 | 2.1 (0.6-3.6) | 1/23 | 4.3 | 0.09 | |
| ASKO | 1 | 0.2 (-0.1-0.5) | 0/1 | 0.0 | 0.00 | | 1 | 0.2 (-0.1-0.5) | 0/1 | 0.0 | 0.00 | 2 | 0.2 (0.0-0.4) | 0/2 | 0.0 | 0.00 | |
| YADI | 7 | 1.2 (0.6-1.7) | 1/6 | 16.7 | 0.19 | | 4 | 0.7 (-0.2-1.6) | 1/3 | 33.3 | 0.22 | 11 | 0.9 (0.5-1.3) | 2/9 | 22.2 | 0.21 | |
| LOMO | 12 | 2.0 (0.5-3.5) | 0/11 | 0.0 | 0.00 | | 13 | 2.2 (1.1-3.3) | 0/7 | 0.0 | 0.00 | 25 | 2.1 (0.9-3.3) | 0/18 | 0.0 | 0.00 | |
| AAW | 86 | 14.3 (6.4-22.3) | 4/51 | 7.8 | 1.12 | | 51 | 8.5 (4.5-12.5) | 0/41 | 0.0 | 0.00 | 137 | 11.4 (6.0-16.8) | 4/92 | 4.3 | 0.56 | |
| KOUB | 118 | 19.7 (12.9-26.4) | 3/102 | 2.9 | 0.58 | | 70 | 11.7 (5.3-18.0) | 0/57 | 0.0 | 0.00 | 188 | 15.7 (11.4-20.0) | 3/159 | 1.9 | 0.29 | |
| MINA | 10 | 1.7 (-0.6-4.0) | 1/7 | 14.3 | 0.24 | | 6 | 1.0 (0.2-1.8) | 0/5 | 0.0 | 0.00 | 16 | 1.3 (0.3-2.3) | 1/12 | 8.3 | 0.12 | |
| NGAH | 4 | 0.7 (-0.2-1.6) | 0/3 | 0.0 | 0.00 | | 7 | 1.2 (-0.6-2.9) | 0/7 | 0.0 | 0.00 | 11 | 0.9 (-0.4-2.2) | 0/10 | 0.0 | 0.00 | |
| KOSS | 1 | 0.2 (-0.1-0.5) | 0/1 | 0.0 | 0.00 | | 2 | 0.3 (0.0-0.7) | 0/2 | 0.0 | 0.00 | 3 | 0.3 (0.0-0.5) | 0/3 | 0.0 | 0.00 | |

HBR: human biting rate expressed as number of b/p/n; SIR: sporozoite infection rate expressed in percent; EIR: entomological rate expressed as number of infected bites/p/n; CI: confidence interval; N: number of mosquitoes collected; Infect./Tested: number of mosquitoes found infected with *Plasmodium* spp. over the total number of mosquitoes tested

Cluster: AAW, Amanzi-Abrika-Wuakre; AHOU, Ahougnansou N’Ganou; AKOI, Akoi N’Denou; ALLA, Allahakoffikro; ASGO, Asse N’Gou; ASKO, Assuikro-Konankuikro; ASMB, Asse M’Bo; ASNG, Asse N’Gattakro; ASSA, Assabonou; AYAP, Ayaprikro; BOFI, Bofia; BONG, Bongobo; BOZA, Bomizambo; DUKP, Duibo-Kpato; GALB, Galebo; GBEG, Gbegbessou; KOMO, Komorossou; KONA, Konankro; KONG, Kongonou Ancien-Kongonou Nouveau; KOSS, Kossou; KOUB, Koubi; LOMO, Lomokankro; MBOU, M’Bouedio; MINA, Minambo; NGAH, N’Gangoro Ahitou-Kpassanou; NGFO, N’Gangoro Nanafoue; NGOI, N’Goimbo; NNAT, N’Gatta N’Guessanblekro-Attienkoffikro; NZIS, N’Zissiessou; PROP, Proponou; TOLLA, Tollabonou-Mekoinkro; TOTO, Totokro; YADI, Yadibikro
